# Efficient management strategy of COVID-19 patients based on cluster analysis and clinical decision tree classification

**DOI:** 10.1038/s41598-021-89187-3

**Published:** 2021-05-05

**Authors:** Zhi Li, Ling Wang, Lv-shuai Huang, Meng Zhang, Xianhua Cai, Feng Xu, Fei Wu, Honghua Li, Wencai Huang, Qunfang Zhou, Jing Yao, Yong Liang, Guoliang Liu

**Affiliations:** 1 Department of Orthopedics, General Hospital of Chinese PLA Central Theater Command, Wuhan, 430070 China; 2 Southern Medical University, Guangzhou, 510515 China; 3 Hubei Key Laboratory of Environmental and Health Effects of Persistent Toxic Substances, Institute of Environment and Health, Jianghan University, Wuhan, 430056 China; 4 Department of Scientific Research Training, General Hospital of Chinese PLA Central Theater Command, Wuhan, 430070 China; 5 Department of Neurology, General Hospital of Chinese PLA Central Theater Command, Wuhan, 430070 China; 6 Department of Radiology, General Hospital of Chinese PLA Central Theater Command, Wuhan, 430070 China; 7 State Key Laboratory of Environmental Chemistry and Ecotoxicology, Research Center for Eco-Environmental Sciences, Chinese Academy of Sciences, Beijing, 100085 China; 8 Department of Pulmonary and Critical Care Medicine, Center of Respiratory Medicine, China-Japan Friendship Hospital; Institute of Respiratory Medicine, Chinese Academy of Medical Sciences, National Clinical Research Center for Respiratory Diseases, Beijing, 100029 China

**Keywords:** Health care, Risk factors

## Abstract

Early classification and risk assessment for COVID-19 patients are critical for improving their terminal prognosis, and preventing the patients deteriorate into severe or critical situation. We performed a retrospective study on 222 COVID-19 patients in Wuhan treated between January 23rd and February 28th, 2020. A decision tree algorithm has been established including multiple factor logistic for cluster analyses that were performed to assess the predictive value of presumptive clinical diagnosis and features including characteristic signs and symptoms of COVID-19 patients. Therapeutic efficacy was evaluated by adopting Kaplan–Meier survival curve analysis and cox risk regression. The 222 patients were then clustered into two groups: cluster I (common type) and cluster II (high-risk type). High-risk cases can be judged from their clinical characteristics, including: age > 50 years, chest CT images with multiple ground glass or wetting shadows, etc. Based on the classification analysis and risk factor analysis, a decision tree algorithm and management flow chart were established, which can help well recognize individuals who needs hospitalization and improve the clinical prognosis of the COVID-19 patients. Our risk factor analysis and management process suggestions are useful for improving the overall clinical prognosis and optimize the utilization of public health resources during treatment of COVID-19 patients.

## Introduction

Coronavirus disease 2019 (COVID-19) pandemic is still spreading worldwide and more than 140 million infected cases have been reported^1^. Currently, COVID-19 cases can be confirmed by the clinical features, including (1) common clinical features like fever, cough, fatigue, dyspnea and anorexia; (2) recent exposure history including clustering onset, residency or a travel history to affected geographic areas and a close contact with suspected or laboratory-confirmed COVID-19 patients within the last 14 days; (3) chest CT abnormality; (4) a positive result of SARS-CoV-2 virus nucleic acid testing^2–6^.

Previous studies of COVID-19 were focused on the patients with positive nucleic acid testing result and hospitalized patients with pneumonia^7,8^. Noteworthily, increasing numbers of patients with pneumonia and similar clinical features while negative nucleic acid testing result were reported in epidemic areas like Wuhan. These patients presented identical clinical processes and poor therapeutic effect with COVID-19, and were finally confirmed as COVID-19 cases even without nucleic acid test. These cases were also excluded from the research of COVID-19 disease, which could not well recognize the disease and might cause higher mortality in treatment of the COVID-19 patients, and part of these patients could not get a good therapeutic effect under the same clinical treatment methods. Furthermore, significant difference and deviation, high false negative rate and false positive rate occurred in the existing SARS-CoV-2 virus nucleic acid testing methods and testing reagents, leading to the fuzzy statistical result of the true positive rate^9^. The objective clinical features are relatively stable in different individuals, and has been proved to help prediction of the prognosis of various diseases. Therefore, though nucleic acid test is crucial for confirming the infection of SARS-CoV-2 virus, the objective clinical features of COVID-19 patients should be more convincing.

According to the clinical data, the estimated incubation time for COVID-19 is 4 days (interquartile range: 2–7 days), 81% of the COVID-19 patients have uncomplicated or mild illness, 19% of them might develop severe or critical illness^1,10,11^. Patients with older age and comorbidities were proved to be at great risk of developing into severe or critical situation even death^11,12^. Heterogeneity of the COVID-19 disease was reported, COVID-19 patients can present distinct prognosis following the treatment. However, factors associated to the different prognosis of COVID-19 patients and clinical judgment of severe or critical cases at early stage is still unclear, which could not be solved by current diagnosis and treatment guidance for COVID-19 disease. Therefore, it is urgent to establish effective clinical pathways and processes for clinical classification of the cases, to distinguish the severe and critical ones at early stage in confirmed and suspected cases, identify and prevent the deterioration of the COVID-19 disease.

In current research, we performed a retrospective study for classification of confirmed COVID-19 cases with similar early clinical features in Wuhan, including both nucleic acid positive and negative cases. Though the true positive and negative rate of these cases were not confirmed, we consider that these patients can fully display the overview of COVID-19 disease. Comparing with the previous studies, here we studied the different disease processes and prognosis of COVID-19 patients and gave clinical classifications for these cases base on the objective clinical features. We further concluded an efficient work chart for prompt diagnosis and appropriate management of COVID-19 patients. Our research can offer identifying method and clear treatment process for general COVID-19 patients, distinguishing cases who might deteriorate into severe or critical situation and improving their terminal prognosis.

## Methods

### Data collection

All the data are collected from infected COVID-19 patients admitted to General Hospital of Chinese PLA Central Theater Command between January 23rd, 2020 and February 28th, 2020, who are ordinary citizens in Wuhan and has been cured or died after clinical treatment. These patients were confirmed to be infected by positive nucleic acid test or clinical diagnosis. All the cases were negative for respiratory virus including respiratory syncytial virus and influenza viruses, etc. The study was approved by the General Hospital of Chinese PLA Central Theater Command Ethics Committee. All methods were performed in accordance with the relevant guidelines and regulations. Since this is a retrospective study need for informed consent was waived by General Hospital of Chinese PLA Central Theater Command Ethics Committee.

The collected data included basic information, clinical symptoms, course of disease, comorbidities, chest CT scanning presentations, first blood test results, initial outcomes (cured, aggravation or death), and final outcomes (cured or death) using standard case report forms. Clinical clarifications of the database were performed by cluster analysis method based on the clinical objective indexes of the patients including gender, age, course of disease, comorbidities, clinical symptoms and chest CT images.

Prognosis of the patients were clarified into early and terminal prognosis. Early prognosis is defined as the status of all patients at the time of their first disease transition, including cured, aggravation or death. Terminal prognosis is defined as the terminal prognosis of all patients, including cured and dead. Acute exacerbation was defined as gradually exacerbation in sequence of mild, common, severe, or critically ill. Respiratory failure or death of mild cases or common cases after three days of hospitalization was also defined as acute exacerbation.

### Statistical analysis

Cluster analysis was used to explore the influencing factors and clinical typing of disease prognosis. Survival analysis and cox regression analysis were performed to evaluate the effects of treatment interventions and the associated risks of prognosis. K-means cluster analysis method was adopted for the cluster analysis, data processing and calculation were performed in SPSS statistical software version 26.0 (IBM Corp, Armonk, NY, USA, 2011). The decision tree model was built by adopting exhaustive CHAID method (exhaustive chi squared automatic interaction) and validated by confusion matrix analysis. Counting data were expressed as percentages, and measurement data were expressed as mean ± standard deviation (SD). Chi-square test and Fisher-exact test were used to compare the difference among the counting data. Independent sample *t* test was used for analysis of measurement data, p < 0.05 was considered statistically different.

## Results

**Figure 1:**
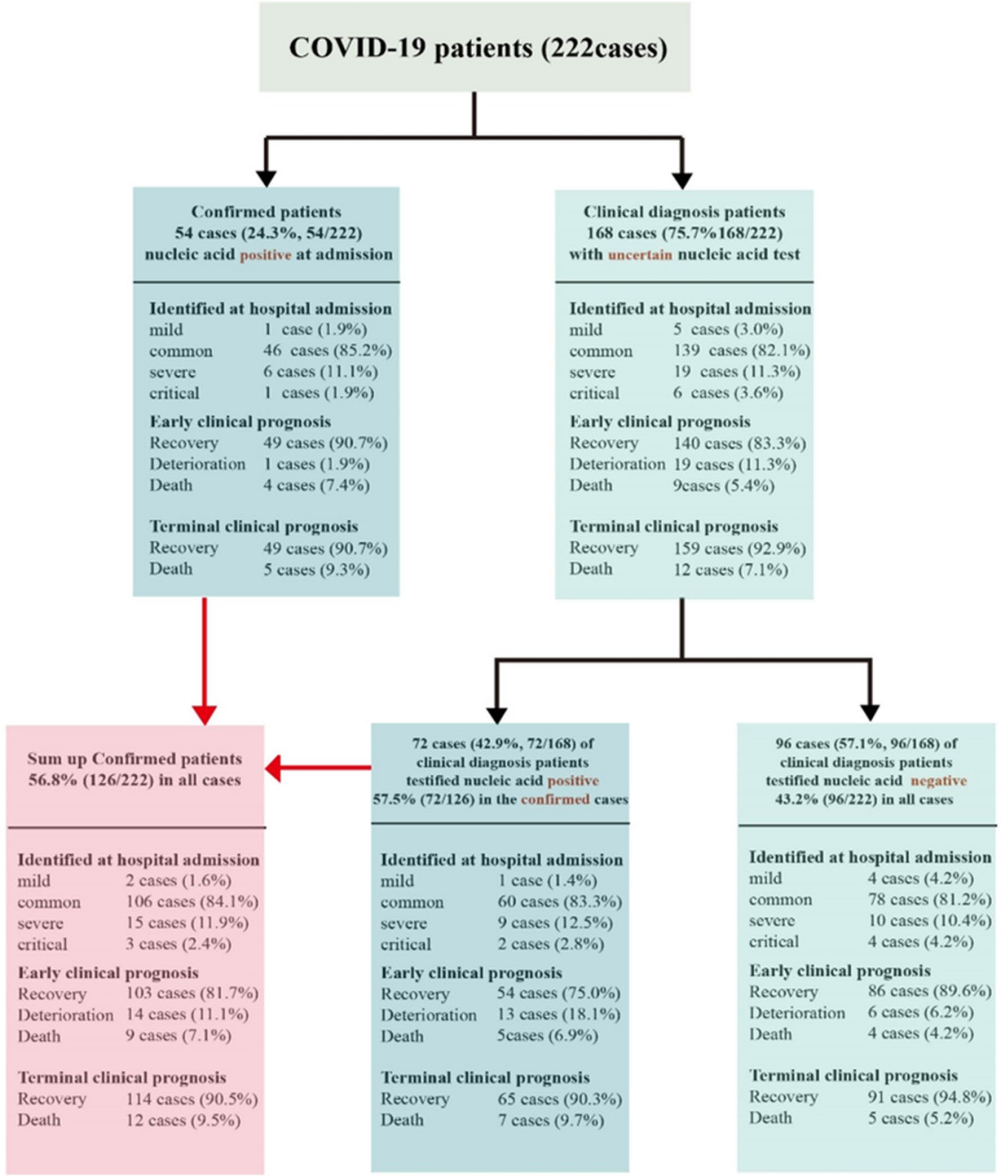
Clinical diagnosis and prognosis of 222 patients.

222 confirmed COVID-19 cases were admitted in this study, their clinical features are listed in Table 1 and Fig. 1. According to the final outcome, all the cases were divided into two groups (recovery group and death group), and their clinical features were compared (Table 1). These data also demonstrated that clinical diagnosis other than nucleic acid testing only is essential for confirming COVID-19 disease. Epidemic history, objective clinical features and chest CT manifestations should be primarily considered for timely treatment of COVID-19. No significant difference occurred between the two groups (Chi square 0.020, P = 0.887), suggesting that the occurrence time of negative nucleic acid test result has little effect on the final prognosis of COVID-19 patients. Therefore, the main goal of treatment for COVID-19 patients should not be nucleic acid negative only.

**Table 1:** Clinical features for 222 COVID-19 patients of different terminal prognosis groups.

Patients’ characteristics	Total (N = 222)	Recovery (N = 205)	Death (n = 17)	P value
Age (years), mean ± SD	50.5 ± 17.9	48.6 ± 16.3	74.5 ± 18.7	0.001
Sex (% male)	102 (45.9)	90 (43.9)	12 (70.6)	0.034
**Course of disease (days)**				
Mean ± SD	8.7 ± 5.8	8.7 ± 5.7	8.9 ± 6.8	0.900
Median	8.0	8.0	7.0	
**Current smoker**	5 (2.3)	2 (10)	3 (17.6)	0.003
**Chronic comorbidities**				
No comorbidity	154 (69.4)	147 (71.7)	7 (41.2)	0.001
One comorbidity	46 (20.7)	43 (21.0)	3 (17.6)	
More than one comorbidity	22 (9.9)	15 (7.3)	7 (41.2)	
Hypertension	37 (16.7)	32 (15.6)	5 (29.4)	0.142
Coronary heart disease	14 (6.3)	9 (4.4)	5 (29.4)	0.001
Diabetes, type 2	19 (8.6)	15 (7.3)	4 (23.5)	0.022
Chronic obstructive lung disease	3 (1.4)	3 (1.5)	0 (0.0)	0.616
Carcinoma	2 (0.9)	2 (1.0)	0 (0.0)	0.218
Cerebral infarction	4 (1.8)	3 (1.5)	1 (5.9)	0.188
Chronic kidney disease	4 (1.8)	0 (0.0)	4 (3.8)	0.047
Gastroesophageal reflux disease	3 (1.4)	3 (1.5)	0 (0.0)	0.616
**Signs and symptoms**				
No symptoms	1 (0.45)	1 (0.5)	0 (0.0)	0.304
One symptoms	16 (7.2)	16 (7.8)	0 (0.0)	
Two symptoms	44 (19.8)	40 (19.5)	4 (23.5)	
Three symptoms	50 (22.5)	48 (23.4)	2 (11.8)	
Four symptoms	43 (19.4)	41 (20.0)	2 (11.8)	
More than four symptoms	68 (30.6)	59 (28.8)	9 (52.9)	
General symptoms				
Fever (temperature ≥ 37·3 °C)	193 (86.9)	178 (86.8)	15 (88.2)	0.869
Chills	19 (8.6)	18 (8.8)	1 (5.9)	0.681
Fatigue	107 (48.2)	99 (48.3)	8 (47.1)	0.922
Anorexia	51 (23.0)	42 (20.5)	9 (52.9)	0.002
Head and neck symptoms				
Rhinorrhoea	10 (4.5)	9 (4.4)	1 (5.9)	0.776
Pharyngalgia	23 (10.4)	23 (11.2)	0 (0.0)	0.228
Chest symptoms				
Chest pain	9 (4.1)	8 (3.9)	1 (5.9)	0.691
Chest tightness	51 (23.0)	48 (23.4)	3 (17.6)	0.587
Dry cough	90 (40.5)	80 (39.0)	10 (58.8)	0.110
Short breath	47 (21.2)	43 (21.0)	4 (23.5)	0.804
Dyspnea	7 (3.2)	6 (2.9)	1 (5.9)	0.503
Expectoration	62 (27.9)	55 (26.8)	7 (41.2)	0.205
Abdominal symptom				
Diarrhea	38 (17.1)	36 (17.8)	2 (11.8)	0.542
Abdominal pain	7 (3.2)	5 (2.4)	2 (11.8)	0.034
Nausea or vomiting	19 (8.6)	15 (7.4)	4 (23.6)	0.289
Nervous system symptoms				
Headache	29 (13.1)	26 (12.7)	3 (17.6)	0.559
Dizziness	5 (2.3)	5 (2.4)	0 (0.0)	0.515
Musculoarticular symptoms				
Arthralgia	11 (5.0)	10 (4.9)	1 (5.9)	0.855
Myalgia	57 (25.7)	52 (25.4)	5 (29.4)	0.714
**Chest CT findings**				
Multiple small patchy shadow	142 (64.0)	140 (68.3)	2 (11.8)	0.001
Multiple ground glass shadow or infiltrative shadow	84 (37.8)	69 (33.7)	15 (88.2)	0.001
Interstitial change	1 (0.5)	0 (0.0)	1 (5.9)	0.001
Pulmonary consolidation	6 (2.7)	5 (2.4)	1 (5.9)	0.400
Pleural effusion	8 (3.6)	4 (2.0)	4 (7.7)	0.001
**Clinical syndromes on admission**				
Mild illness	6 (2.7)	6 (2.9)	0 (0.0)	0.001
Pneumonia	184 (82.9)	180 (87.8)	4 (23.5)	
Severe pneumonia	25 (11.3)	19 (9.3)	6 (35.3)	
Critical pneumonia	7 (3.2)	0 (0.0)	7 (41.2)	
**Diagnosis**				
Confirmed cases with positive nucleic acid testing result on admission	54 (24.3)	49 (23.9)	5 (29.4)	0.611
Clinically confirmed cases on admission	168 (75.7)	156 (76.1)	12 (70.6)	
SARS-CoV-2 nucleic acid positive cases	126 (56.8)	114 (55.6)	12 (70.6)	0.231
SARS-CoV-2 nucleic acid negative cases	96 (43.2)	91 (44.4)	5 (29.4)	
Time from onset to first positive nucleic acid test (days)	8.9 ± 5.7	9.10 ± 5.9	8.3 ± 4.2	0.566
Time from onset to first negative nucleic acid test (days)	16.81 ± 8.3	16.9 ± 8.2	14.00 ± 14.5	0.887
**Treatment**				
Nasal catheter/mask oxygen therapy on admission	174 (78.4)	160 (78.0)	14 (82.4)	0.679
High flow nasal catheter oxygen therapy	12 (5.4)	6 (2.9)	6 (35.3)	0.001
Mechanical ventilation				
Non-invasive	19 (8.6)	3 (1.5)	16 (94.1)	0.001
Invasive	6 (2.7)	0 (0.0)	6 (35.3)	0.001
Extracorporeal membrane oxygenation	2 (1.0)	0 (0.0)	2 (11.8)	0.635
Antibacterial agents	201 (90.5)	184 (89.8)	17 (100)	0.165
Glucocorticoids	102 (45.9)	89 (43.4)	13 (76.5)	0.009
Antiviral agents	209 (94.1)	193 (94.1)	16 (94.1)	0.996
Oseltamivir	180 (81.1)	170 (82.9)	10 (58.8)	0.015
Interferon	106 (47.7)	100 (48.8)	6 (35.3)	0.285
Lopinavir and ritonavir	73 (32.9)	65 (31.7)	8 (47.1)	0.195
Ribavirin	77 (34.7)	73 (35.6)	4 (23.5)	0.315
Abidol	16 (7.2)	13 (6.3)	3 (17.6)	0.083
Immunoenhancer	160 (72.1)	144 (70.2)	16 (94.1)	0.035
Thymosin	122 (55.0)	110 (53.7)	12 (7.7)	0.178
Immunoglobulin	131 (59.0)	115 (56.1)	16 (94.1)	0.002

**Figure 2:**
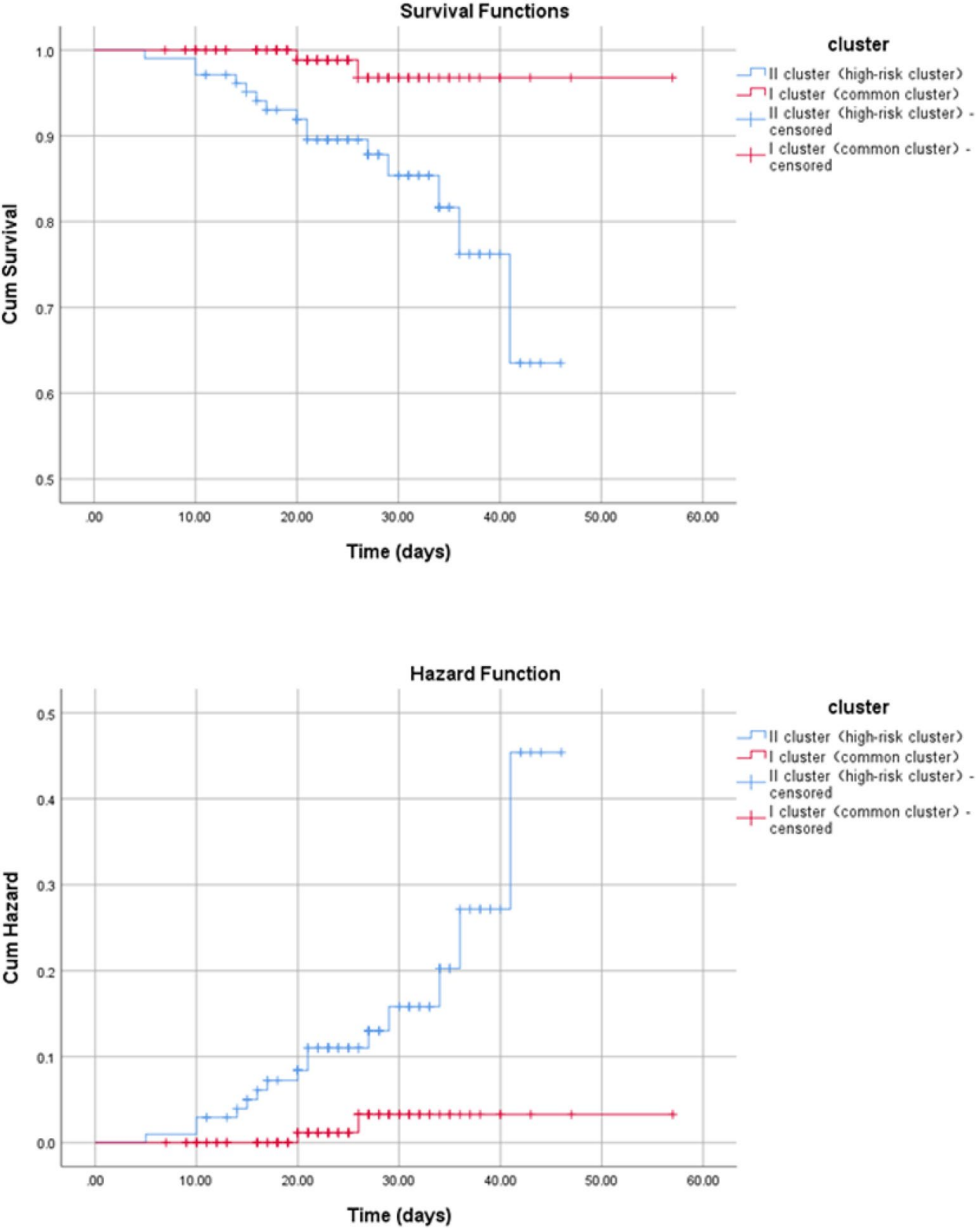
Kaplan–Meier survival curve analysis for two types of COVID-19 patients.

The following factors were finally applied in cluster analysis : (1) age of 50 years; (2) comorbidities including smoking, diabetes, hypertension, coronary heart disease, cerebral infarction, chronic renal failure; (3) clinical symptoms including cough, fatigue, anorexia chest tightness; (4) chest CT manifestation like multiple small patchy shadows, multiple ground glass shadow or infiltrating shadow. The 222 patients were then divided into two groups (Table 2 and Table S1). Based on the clinical characteristics and prognosis, the two groups were named as cluster I (common type) and cluster II (high-risk type). As depicted in Fig. 2, the mean survival time for cluster II patients was 40.4 days (95% CI 37.8–43.0 days), which was significantly shorter (Kaplan–Meier survival curve analysis, chi square 8.873, P = 0.003) than that for cluster I patients (55.1 days, 95% CI 54.4–57.4 days). The main clinical features of cluster II patients were age > 50 years, cough, fatigue, anorexia and chest CT images with multiple ground glass or infiltrates (Table 2). Other typical clinical features of cluster II patients include: comorbidities like smoking, diabetes, hypertension, coronary heart disease, cerebral infarction and chronic renal failure; hyper-inflammatory state occurred in these patients (Table 2 and Table S1).

**Table 2:** Clinical features and clinical clusters of 222 COVID-19 patients.

Clinical characteristics of the patients	Total (N = 222)	Cluster I (common type, N = 118)	Cluster II (high-risk type, N = 104)	P value
Age (years), mean ± SD	50.5 ± 17.9	36.6 ± 8.9	66.3 ± 11.1	0.691
Sex (% male)	102 (45.9)	57 (48.3)	43.3 (44.1)	0.501
**Course of disease** (**days)**				
Mean ± SD	8.7 ± 5.8	7.9 ± 5.5	9.6 ± 6.0	0.020
Median	8.0	7.0	9.0	
Current smoker	5 (2.3)	0 (00)	5 (4.8)	0.021
**Chronic comorbidities**				
No comorbidities	154 (69.4)	106 (89.8)	48 (46.2)	0.001
One comorbidity	46 (20.7)	11 (9.3)	35 (33.7)	
More than one comorbidities	22 (9.9)	1 (0.8)	21 (20.2)	
Hypertension	37 (16.7)	17 (5.9)	30 (28.8)	0.001
Coronary heart disease	14 (6.3)	0 (0.0)	14 (13.5)	0.001
Diabetes, type 2	19 (8.6)	2 (1.7)	17 (16.3)	0.001
Chronic obstructive lung disease	3 (1.4)	0 (0.0)	3 (2.9)	0.101
Carcinoma	2 (0.9)	0 (0.0)	2 (1.9)	0.218
Cerebral infarction	4 (1.8)	0 (0.0)	4 (3.8)	0.047
Chronic kidney disease	4 (1.8)	0 (0.0)	4 (3.8)	0.047
Gastroesophageal reflux disease	3 (1.4)	1 (0.8)	2 (1.9)	0.601
**Signs and symptoms**				
No symptom	1 (0.5)	0 (0.0)	1 (1.0)	0.179
One symptom	16 (7.2)	12 (10.2)	4 (3.8)	
Two symptoms	44 (19.8)	27 (22.9)	17 (16.3)	
Three symptoms	50 (22.5)	24 (20.3)	26 (25.0)	
Four symptoms	43 (19.4)	24 (20.3)	19 (18.3)	
More than four symptoms	68 (30.6)	31 (26.3)	37 (35.6)	
General symptoms				
Fever (temperature ≥ 37.3 °C)	192 (86.5)	107 (90.7)	85 (81.7)	0.075
Chills	19 (8.6)	8 (6.8)	11 (10.6)	0.345
Fatigue	107 (48.2)	47 (39.8)	60 (57.7)	0.008
Anorexia	51 (23.0)	19 (16.1)	32 (30.8)	0.011
Head and neck symptoms				
Rhinorrhoea	10 (4.5)	6 (5.1)	4 (3.8)	0.753
Pharyngalgia	23 (10.4)	17 (12.7)	8 (7.7)	0.272
Chest symptoms				
Chest pain	9 (4.1)	4 (3.4)	5 (4.8)	0.737
Chest tightness	51 (23.0)	25 (21.2)	26 (25.0)	0.500
Dry cough	90 (40.5)	36 (30.5)	54 (51.9)	0.002
Short breath	47 (21.2)	26 (22.0)	21 (20.2)	0.738
Dyspnea	7 (3.2)	2 (1.7)	5 (4.8)	0.257
Expectoration	62 (27.9)	27 (22.9)	35 (33.7)	0.099
Abdominal symptom				
Diarrhea	37 (16.7)	21 (17.8)	16 (15.4)	0.719
Abdominal pain	7 (3.2)	5 (4.2)	2 (1.9)	0.452
Nausea or vomiting	19 (8.6)	7 (5.9)	13 (11.5)	0.010
Nervous system symptoms				
Headache	29 (13.1)	20 (16.9)	9 (8.7)	0.075
Dizziness	5 (2.3)	5 (4.2)	0 (0.0)	0.062
Musculoarticular symptoms				
Arthralgia	11 (5.0)	5 (4.2)	6 (5.8)	0.600
Myalgia	57 (25.7)	29 (24.6)	28 (26.9)	0.759
**Chest CT findings**				
Multiple small patchy shadow	142 (64.0)	85 (72)	57 (54.8)	0.008
Multiple ground glass shadow or infiltrative shadow	84 (37.8)	33 (28)	51 (49.8)	0.001
Interstitial change	1 (0.5)	0 (0.0)	1 (1.0)	0.468
Pulmonary consolidation	6 (2.7)	4 (3.4)	2 (1.9)	0.687
Pleural effusion	8 (3.6)	1 (0.8)	7 (6.7)	0.027
**Clinical syndromes on admission**				
Mild illness	6 (2.7)	5 (4.2)	1 (1.0)	0.003
Asymptomatic infection	1 (0.50	1 (0.8)	0 (0.0)	
Pneumonia	184 (82.9)	104 (88.1)	80 (76.9)	
Severe pneumonia	25 (11.3)	9 (7.6)	16 (15.4)	
Critical pneumonia	7 (3.2)	0 (0.0)	7 (6.7)	
**Diagnosis**				
Confirmed cases with positive nucleic acid test result on admission	54 (24.3)	31 (26.3)	23 (22.1)	0.53
Clinically confirmed cases on admission	168 (75.7)	87 (73.7)	81 (77.9)	
SARS-CoV-2 nucleic acid positive cases	126 (56.8)	71 (60.2)	55 (52.9)	0.281
SARS-CoV-2 nucleic acid negative cases	96 (43.2)	47 (39.8)	49 (47.1)	
**Treatment**				
Nasal catheter/mask oxygen therapy on admission	174 (78.4)	89 (75.4)	85 (81.7)	0.327
High flow nasal catheter oxygen therapy	12 (5.4)	3 (2.5)	9 (8.7)	0.071
Mechanical ventilation				
Non-invasive	19 (8.6)	4 (3.4)	15 (14.4)	0.004
Invasive	6 (2.7)	1 (0.8)	5 (4.8)	0.101
Extracorporeal membrane oxygenation	2 (1.0)	1 (0.8)	2 (2.0)	0.368
Antibacterial agents	201 (90.5)	09 (92.4)	92 (88.5)	0.363
Glucocorticoids	102 (45.9)	47 (39.8)	55 (52.9)	0.059
Antiviral agents	209 (94.1)	111 (94.1)	98 (94.2)	0.959
Oseltamivir	180 (81.1)	98 (83.1)	82 (78.8)	0.493
Interferon	106 (47.7)	56 (47.5)	50 (48.1)	0.927
Lopinavir and ritonavir	73 (32.9)	44 (37.3)	29 (27.9)	0.154
Ribavirin	77 (34.7)	48 (40.7)	29 (27.9)	0.049
Abidol	16 (7.2)	8 (6.8)	8 (7.7)	0.801
Immunoenhancer	160 (72.1)	79 (66.9)	81 (77.9)	0.074
Thymosin	122 (55.0)	66 (55.9)	56 (53.8)	0.788
Immunoglobulin	131 (59.0)	60 (50.8)	71 (68.3)	0.010
**Early prognosis**				
Recovery	189 (85.1)	107 (90.7)	82 (78.8)	0.003
Aggravation	20 (9.0)	10 (8.5)	10 (9.6)	
Death	13 (5.9)	1 (0.8)	12 (11.5)	
**Terminal prognosis**				
Recovery	205 (92.3)	116 (98.3)	89 (85.6)	0.001
Death	17 (7.7)	2 (1.7)	15 (14.4)

According to logistic regression analysis data, shortness of breath, smoking, diabetes, hypertension and coronary heart disease, multiple ground glass or infiltrative shadow on chest CT were the risk factors for patients to develop into severe or critical diseases, while the degree of disease was not related to age, fever or positive nucleic acid test results (Table S2). Two types of CT manifestations were related to prognosis: multiple small patchy shadows, multiple ground glass shadow and infiltrating shadow. Patients with multiple small patches presented a better prognosis, with a lower exacerbation rate and mortality (Table S2). Their estimated mean time of progression to severe disease was 16.9 days (95% CI 13.6–20.2 days), significantly shorter than the 29.8 days (95% CI 29.3–30.2 days) for those without multiple ground glass shadow or infiltrative shadow (Kaplan–Meier survival curve analysis, Chi square 43.687, P = 0.000). All the patients were then clarified into 4 groups according to the chest CT images (Table S3).

Since no specific drug targeting COVID-19 disease has been explored, multiple drugs are applied in treating the patients. Here we performed Kaplan–Meier survival curve analysis and cox risk regression by using the cluster analysis factors, aiming to give a brief evaluation of these drugs (Figure S1 and Table 3). Both nucleic acid negative and positive patients with anorexia presented increased risks of death, which might be improved by using oseltamivir (Table 3). Treatment with lopinavir and ritonavir could reduce the risk of death in all the patients especially in nucleic acid positive patients. Oseltamivir can prolong the survival time of nucleic acid negative patients, and glucocorticoid and immunoglobulin can significantly shorten the survival time of nucleic acid positive patients; while lopinavir and ritonavir could not improve survival in nucleic acid negative or positive patients (Figure S1).

**Table 3:** Cox regression analysis for risk of death following different treatments and final prognosis of COVID-19 patients.

Treatment	Total				SARS-CoV-2 nucleic acid positive patients			
	B	SE	Wald	P	B	SE	Wald	P
Nasal catheter/mask oxygen therapy on admission	− 1.06	1.13	0.87	0.350	− 5.19	134	0.001	0.969
High flow nasal catheter oxygen therapy	2.34	1.12	4.34	0.037	13.0	30.2	0.18	0.668
Non-invasive mechanical ventilation	13.8	52.7	0.07	0.793	35.0	65.2	0.29	0.592
Invasive mechanical ventilation	1.18	1.23	0.92	0.337	2.61	1.42	3.37	0.066
Antibacterial agents	6.84	185	0.001	0.970	− 15.5	73.8	0.04	0.834
Glucocorticoids	2.89	1.19	5.95	0.015	15.7	22.7	0.48	0.489
Oseltamivir	− 0.04	0.97	0.001	0.971	5.49	2.52	4.72	0.030
Lopinavir and ritonavir	− 2.59	1.05	6.05	0.014	− 9.71	3.69	6.93	0.008
Interferon	0.58	0.88	0.43	0.514	1.95	1.50	1.69	0.194
Ribavirin	− 1.74	1.34	1.70	0.192	− 1.66	1.44	1.33	0.249
Abidol	− 0.06	1.20	0.002	0.961	1.80	1.65	1.18	0.277
Thymosin	1.04	1.14	0.83	0.363	2.18	1.62	1.81	0.178
Immunoglobulin	− 9.25	52.7	0.03	0.861	− 13.2	129	0.01	0.918

According to the conclusions obtained above, we established a decision tree for determining the severe or critical cases by combining our clinical experiences with the analysis of factors involved in acute exacerbation and risk of death for patients after the early prognosis (Figure S2 and Figure S3). According to this tree classification model, 90.1% of the patients with risk of acute exacerbation and death (risk probability 9.9%) might occur after early prognosis (Figure S2). In addition, we also build a decision tree model without chest CT results by using short breath, fever, number of comorbidities and age (> 50 years) as independent variables (Figure S3). This model can predict 86% of the patients with risk of acute exacerbation and death (14% of risk probability) after early prognosis. Confusion matrix analysis was also employed to validate the decision tree model (Tables S4–S7).

**Figure 3:**
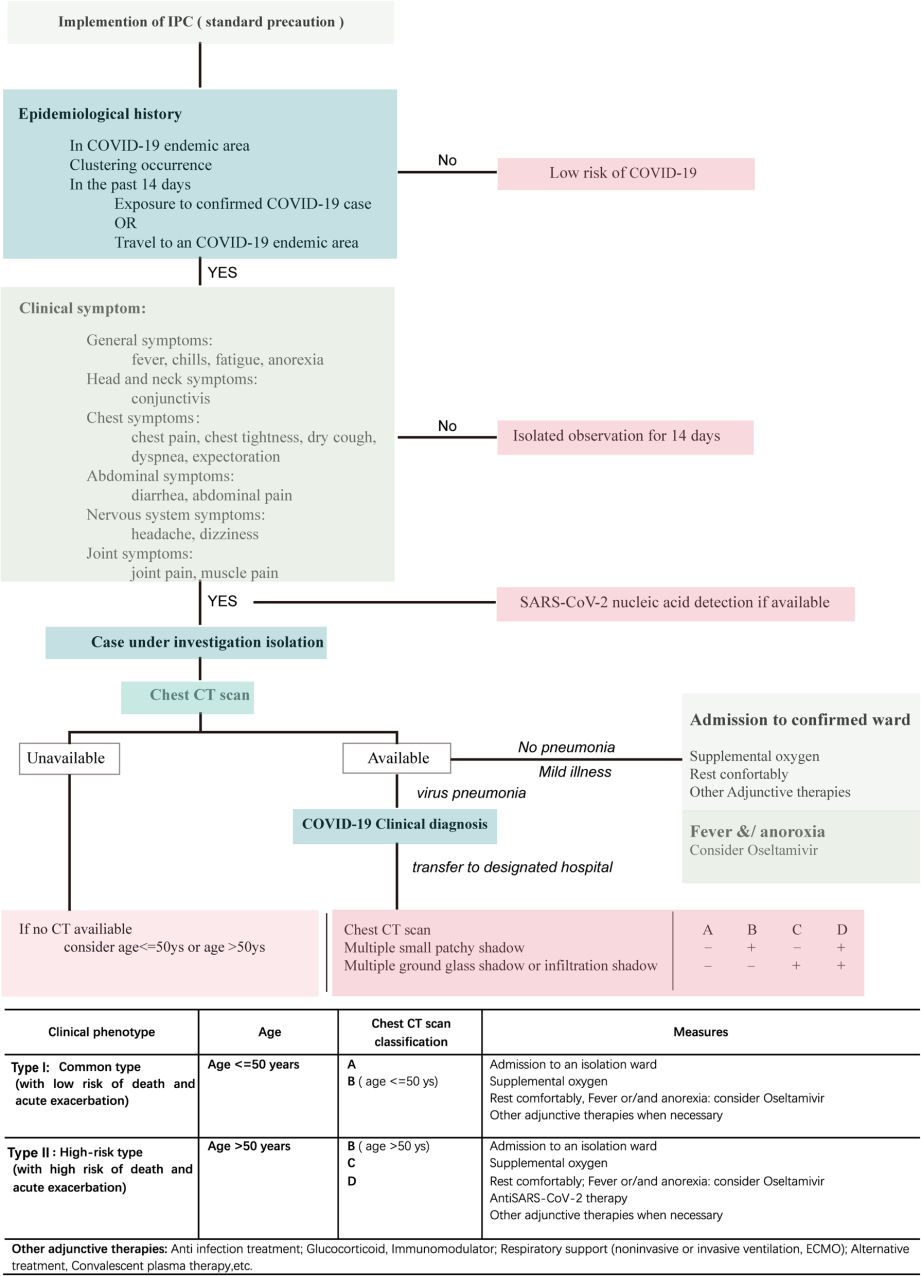
Proposed flow chart for treatment of general COVID-19 patients.

**Figure 4:**
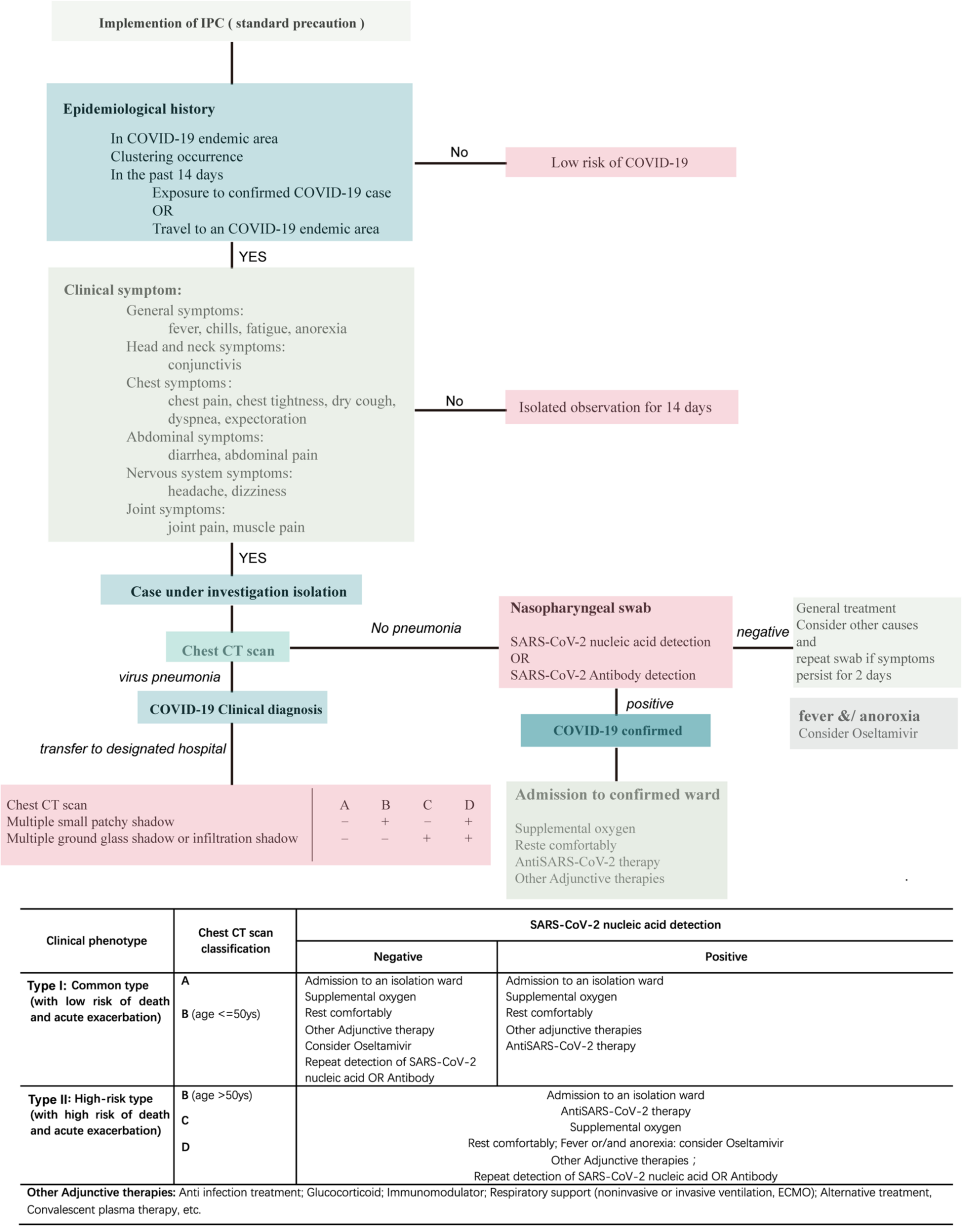
Proposed flow chart for treatment of COVID-19 patients in areas with limited medical resources.

Finally, we suggest an efficiency therapeutic scheme for treatment of COVID-19 patients in general areas (Fig. 3) and areas with limited medical resources (Fig. 4). According to the two flow charts, epidemiological history of patients was primarily considered and followed by their clinical symptoms. For the confirmed cases, chest CT scan is then suggested for pneumonia examination. In particular, for patients in areas with limited medical resources where chest CT examination is unavailable, age is recommended as the key indicator (Fig. 4). Patients without pneumonia should be checked by further nucleic acid test or anti-body test, which is essential for subsequent treatment (Fig. 3). General treatment should be applied for SARS-CoV-2 virus positive patients. These tests can be left out in areas with limited medical resources (Fig. 4). Combining the clinical diagnosis and CT images, patients with pneumonia can be clarified into 4 groups (A, B, C and D). Detailed medical therapy for these 4 groups of patients can further confirmed by classification of their clinical phenotypes (Figs. 3 and 4). Specifically, these patients can be divided into two types (common type and high-risk type) after clustering analysis by using chest CT manifestation, negative clinical features such as age and comorbidities. Consequently, these patients can be treated timely according to their appropriate therapies, which is important before the confirmation of nucleic acid testing result.

## Discussion

Positive SARS-CoV-2 nucleic acid test is now considered crucial for confirming a COVID-19 case. However, a large number of nucleic acid negative patients with epidemiological history, same clinical manifestations and chest CT performance of COVID-19 existed in the endemic area, which was neglected in the initial COVID-19 treatment plan. In considering the uncertain false negative rate of nucleic acid test and unascertained cause of pneumonia by known viruses or other pathogens, these cases were included in our study, which was critical for diagnosis of COVID-19 disease by referring to the chest CT and clinical manifestations in epidemic area. Routine testing for non-SARS-CoV-2 respiratory pathogens during the COVID-19 pandemic was considered unlikely to provide clinical benefit unless a positive result would change disease management (e.g., neuraminidase inhibitors for influenza in appropriate patients)^13^. All of the patients in current study were confirmed as influenza virus negative, and their symptoms could not be alleviated by anti-influenza drug abidor, thus they cannot simply be considered as a patient with influenza virus-negative influenza pneumonia. Due to the absence of clinical anatomical study for these patients, we speculate that positive nucleic acid test result might not be an early manifestation of COVID-19 patients. Moreover, regarding of the limitations of current nucleic acid detection technology or the characteristics of COVID-19, further research is urgent for the treatment of these patients. Our data also indicated that the clinical response and terminal prognosis of these patients with similar chest CT and objective clinical manifestations was not affected by the time and results of nucleic acid test, or when the nucleic acid test result changed from positive into negative. We thus believe that the objective clinical performance and the final clinical prognosis goals, rather than the nucleic acid negative conversion ratio only, should be considered for effective treatment of the COVID-19 cases. We also suggest that improving the survival rate of the COVID-19 patients, rather than alleviating their clinical symptoms, is crucial to evaluate the therapeutic effects of drugs and treatments.

Our cluster analysis indicated that COVID-19 patients can be divided into groups with different clinical prognosis outcomes based on their chest CT features, objective clinical manifestations and related risk factors. Therefore, classification management of the patients is essential in their isolation protection and clinical treatment due to the heterology of COVID-19. Though a decrease in oxygen saturation is considered as the indicator for severe cases, it is not feasible to effectively identify the severe illness by referring to their oxygen partial pressure and oxygen saturation. Here we found that patients with dry cough, abdominal pain and anorexia might easily develop into severe illness or die. Radiography is essential examination for conformation and early diagnosis of COVID-19 disease^14^. Multiple ground glass shadow and infiltrating shadow may occur before a positive nucleic acid test, and present a good consistency with pathological manifestations^15,16^. Therefore, chest CT manifestations can be used to predict the immune state, pathological and physiological conditions of COVID-19 patients.

Older COVID-19 patients and those with comorbidities have increased risk for severe disease and death^11,17–21^. We also found age could be used for clinical classification and prognosis of the patients in areas without chest CT testing facilities. Nucleic acid positive cases with abdominal pain and anorexia, cases with more than two comorbidities, cases with dyspnea, anorexia, and multiple ground glass shadow or infiltrating shadow in Chest CT images got higher risks for acute aggravation of illness, and needs timely hospital admission. We therefore consider that identifying the objective clinical manifestations of patients is more important for timely management of COVID-19 patients in epidemic area. Corresponding treatment should be performed according to the risk evaluation, regardless of the nucleic acid testing results, which is instructive for mild patients under home quarantine or hospital isolation.

Management of the COVID-19 patients brings great challenges and stress to health-care system of epidemic areas. Therefore, effective clinical pathways and processes for clinical classification of COVID-19 patients are essential to distinguish the severe and critical ones at early stage in confirmed and suspected cases, identify and prevent the deterioration of the COVID-19 patients. Multiple studies have raised factors like comorbidities, inflammatory cytokines and lymphocytes as the predictors of disease severity for COVID-19 patients, which can help to identify the severe and critical cases timely^17–19,21,22^. A recent article suggested to develop and validate a clinical score at hospital admission to predict which patients with COVID-19 will develop critical illness^23^. The flow chart we proposed will help general population to diagnose themselves when get clinical features of COVID-19, also make for timely and efficient treatment of confirmed COVID-19 patients by fast classification.

Our study also has some limitations. (1) Some of the patients presented negative result even after several SARS-CoV-2 virus nucleic acid tests. Since these patients could not be distinguished from nucleic acid positive patients with same clinical features, we decided to include these cases into our study. Though this is close to the real clinical practices, these patients might be misdiagnosed considering the existence of false negative results for known respiratory viruses including influenza viruses, as well as the absence of autopsy study for such cases. (2) Since various methods were used for SARS-CoV-2 virus nucleic acid tests, the positive rate, false negative rate and negative predictive value of the test methods were not effectively evaluated. Although the objective clinical features were emphasized, deviations might also exist during the evaluation of clinical data for these nucleic acid negative patients. We will perform antibody test and further confirm the infection of SARS-CoV-2 virus for these patients in the following clinical practices. (3) Single-center study with fewer mild cases has limited our study, further research will be performed to improve our conclusions.

Innovation of our study include: (1) The clinical classification based on objective clinical manifestations is helpful for early identification of high-risk patients and their further clinical treatment; Epidemic history and objective clinical features of the patients should be considered for early prognosis; (2) High-risk patients can be judged from their clinical characteristics (age > 50 years, chest CT images with multiple ground glass or wetting shadows, etc.); (3) Clinical effects of current treatments were evaluated; (4) The timing and purpose of nucleic acid test is proposed based on prognostic classification; (5) A clear flow chart for efficient management of COVID-19 patients is proposed, which can help get effective allocation of medical resources.

## Supplementary Information

Supplementary Information.
