# Supplementary material for: Efficient management strategy of COVID-19 patients based on cluster analysis and clinical decision tree classification
Source: Sci Rep. 2021 May 5;11:9626. doi: 10.1038/s41598-021-89187-3 (PMC8100107; doi:10.1038/s41598-021-89187-3)
Supplement: Supplementary file 1 — Supplementary Information. [file 41598_2021_89187_MOESM1_ESM.docx]

**Supporting Information**

**Efficient management strategy of COVID-19 patients based on cluster analysis and clinical decision tree classification**

Zhi Li^a, b, #^; Ling Wang^c, #^, Lv-shuai Huang^d^, Meng Zhang^c^, Xianhua Cai^a^, Feng Xu^a^, Fei Wu^e,*^, Honghua Li^e^, Wencai Huang^f^, Qunfang Zhou^g^, Jing Yao^d^, Yong Liang^c,*^, Guoliang Liu^h,*^

a. Department of Orthopedics, General Hospital of Chinese PLA Central Theater Command, 430070, Wuhan, China

b. Southern Medical University, 510515, Guangzhou, China

c. Hubei Key Laboratory of Environmental and Health Effects of Persistent Toxic Substances, Institute of Environment and Health, Jianghan University, 430056, Wuhan, China Clinical Research Center for Respiratory Diseases, 100029, Beijing, China

d. Department of Scientific Research Training, General Hospital of Chinese PLA Central Theater Command, 430070, Wuhan, China

e. Department of Neurology, General Hospital of Chinese PLA Central Theater Command, 430070, Wuhan, China

f. Department of Radiology, General Hospital of Chinese PLA Central Theater Command, 430070, Wuhan, China.

g. State Key Laboratory of Environmental Chemistry and Ecotoxicology, Research Center for Eco-Environmental Sciences, Chinese Academy of Sciences, 100085, Beijing, China

h. Department of Pulmonary and Critical Care Medicine, Center of Respiratory Medicine, China-Japan Friendship Hospital; Institute of Respiratory Medicine, Chinese Academy of Medical Sciences; National Clinical Research Center for Respiratory Diseases, 100029, Beijing, China

# These authors contribute equally to this work.

* Corresponding Authors

Fei Wu, e-mail: bladeflyer@126.com

Yong Liang, e-mail: ly76@263.net

Guoliang Liu, e-mail: Liuguoliang@zryhyy.com.cn

**Table S1 Detailed information for clinical blood indicators of two types of patients**

| **Clinical blood indicators** | **Cluster Ⅰ**  （Common type, N=118） | | **Cluster Ⅱ**  （High-risk type, N=104） | | **P Value** |
| --- | --- | --- | --- | --- | --- |
|  | Mean | SD | Mean | SD |  |
| White blood cell count (× 10^9^/L) | 6.4 | 7.1 | 5.4 | 2.8 | 0.181 |
| Neutrophil percentage, % | 63.8 | 14.3 | 67.9 | 15.6 | 0.041 |
| Lymphocyte percentage, % | 25.9 | 12.0 | 23.2 | 12.6 | 0.111 |
| Lymphocyte count (× 10^9^/L) | 1.23 | 0.54 | 1.07 | 0.58 | 0.039 |
| Eosinophil percentage, % | 0.5 | 0.6 | 0.5 | 1.0 | 0.776 |
| Eosinophils (× 10^9^/L) | 0.03 | 0.06 | 0.03 | 0.05 | 0.780 |
| Hemoglobin (g/L) | 133 | 15 | 125 | 18 | 0.001 |
| Platelet count (× 10^9^/L) | 188 | 57 | 193 | 84 | 0.579 |
| T cells (CD3+CD19-) % | 68.3 | 11.5 | 62.6 | 12.2 | 0.003 |
| T cells (CD3+CD19-) /ul | 910 | 487 | 656 | 423 | 0.001 |
| Ts cells (CD3+CD8+) % | 27.9 | 28.1 | 20.9 | 7.7 | 0.046 |
| Ts cells (CD3+CD8+) /ul | 329 | 165 | 225 | 154 | 0.000 |
| Th cells (CD3+CD4+) % | 35.8 | 9.5 | 36.6 | 10.6 | 0.636 |
| Th cells (CD3+CD4+) /ul | 447 | 275 | 378 | 271 | 0.123 |
| NK cells (CD3-/CD16+CD56+) % | 15.2 | 9.7 | 18.6 | 12.1 | 0.054 |
| NK cells (CD3-/CD16+CD56+) /ul | 190 | 147 | 180 | 140 | 0.680 |
| B cells (CD3-CD19+) % | Death | 17(7.7) | 2(1.7) | 15(14.4) |  |
| B cells (CD3-CD19+) /ul | 174 | 114 | 148 | 98.6 | 0.133 |
| Th/Ts | 1.52 | 0.60 | 2.06 | 1.03 | 0.000 |
| C-reactive protein | 18.1 | 27.0 | 32.1 | 34.3 | 0.001 |
| Procalcitonin | 0.07 | 0.17 | 0.17 | 0.80 | 0.186 |
| Interleukin-6, pg/mL | 15.8 | 15.3 | 37.6 | 62.8 | 0.000 |
| Prothrombin time | 12.5 | 2.9 | 12.8 | 6.6 | 0.653 |
| Prothrombin activity | 84 | 14 | 86 | 18 | 0.326 |
| International standardized ratio,INR | 1.13 | 0.09 | 1.18 | 0.61 | 0.371 |
| Fibrinogen determination | 4.10 | 0.83 | 4.27 | 0.80 | 0.124 |
| Activated partial thromboplastin time | 33.1 | 3.8 | 31.7 | 6.8 | 0.043 |
| Thrombin time determination | 13.2 | 1.1 | 13.7 | 1.7 | 0.012 |
| D-dimer | 165 | 157 | 542 | 1395 | 0.005 |
| Fibrin degradation products | 6.11 | 24.59 | 7.94 | 26.99 | 0.606 |
| Antihemozyme Ⅲ activity | 99.9 | 13.6 | 92.5 | 14.7 | 0.000 |
| Factor Ⅷ activity | 136 | 90.8 | 144 | 57.8 | 0.404 |
| Total bilirubin | 10.9 | 6.3 | 12.9 | 8.0 | 0.041 |
| Direct bilirubin | 3.3 | 3.1 | 3.8 | 2.6 | 0.180 |
| Alanine aminotransferase | 26.6 | 31.3 | 40.9 | 123.3 | 0.229 |
| Glutamic oxaloacetylase | 29.3 | 17.8 | 71.4 | 289.3 | 0.118 |
| Albumin | 41.1 | 6.3 | 36.5 | 5.5 | 0.000 |
| Cholinesterase | 6977 | 2006 | 6234 | 1498 | 0.002 |
| Prealbumin | 0.16 | 0.07 | 1.04 | 9.18 | 0.314 |
| Urea | 4.09 | 3.34 | 6.65 | 9.46 | 0.007 |
| Creatinine | 66.9 | 61.6 | 80.1 | 80.3 | 0.171 |
| Carbon dioxide binding force | 23.0 | 1.8 | 22.1 | 3.9 | 0.030 |
| blood glucose | 6.12 | 1.79 | 8.44 | 7.92 | 0.002 |
| Phosphocreatine kinase | 158 | 231 | 250 | 805 | 0.242 |
| Creatine kinase isoenzyme | 18.7 | 17.6 | 20.5 | 12.8 | 0.409 |
| Lactate dehydrogenase | 206 | 64.7 | 279 | 141 | 0.000 |
| Alpha hydroxybutyrate dehydrogenase | 166 | 55.5 | 235 | 125 | 0.000 |
| CKMB | 1.00 | 1.12 | 2.60 | 4.91 | 0.001 |
| Troponin | 0.006 | 0.006 | 0.874 | 8.663 | 0.294 |
| Myoglobin | 32.3 | 28.5 | 70.7 | 74.7 | 0.000 |
| BNP | 58.1 | 115.4 | 307 | 567 | 0.000 |

**Table S2 Risk factors for deterioration of COVID-19 into severe illness by using Logistic regression analysis**

| **Risk factors** | **B** | **SE** | **Wald** | **Sig.** |
| --- | --- | --- | --- | --- |
| Sex (1=male, 2=Female) | 0.331 | 1.018 | 0.106 | 0.745 |
| Age | 0.041 | 0.029 | 2.020 | 0.155 |
| Fever | 1.763 | 1.331 | 1.754 | 0.185 |
| Short breath | 2.099 | 1.127 | 3.469 | 0.063 |
| Smoking | 6.175 | 2.798 | 4.871 | 0.027 |
| Diabetes,Type 2 | 6.040 | 3.215 | 3.530 | 0.060 |
| Hypertension | 7.207 | 2.699 | 7.132 | 0.008 |
| Coronary heart disease | 5.475 | 2.486 | 4.849 | 0.028 |
| Chest CT: multiple ground glass shadow or infiltrative shadow | 4.712 | 1.947 | 5.859 | 0.·015 |
| SARS-CoV-2 nucleic acid positive | -0.309 | 0.706 | 0.191 | 0.662 |

**Table S3 Details for clinical blood indicators from four types of patients clarified by chest CT images**

|  | **A**  **S (-) G (-)** | | **B**  **S (+) G (-)** | | **C**  **S (+) G (-)** | | | **D**  **S (+) G (-)** | |
| --- | --- | --- | --- | --- | --- | --- | --- | --- | --- |
|  | Mean ± SD | Sig. | Mean ± SD | Sig. | Mean ± SD | Sig. | Mean ± SD | | Sig. |
| C-reactive protein | 17.4 ± 20.8 |  | 17.3 ± 22.2 |  | 40.6 ± 42.2 | A (0.021);  B (0.000) | 25.8 ± 0.50 | |  |
| Procalcitonin | 0.09 ± 0.15 |  | 0.06 ± 0.06 |  | 0.26 ± 1.06 |  | 0.07 ± 0.07 | |  |
| Interleukin-6 | 7.88 ± 7.26 |  | 17.1 ± 17.3 |  | 46.8 ± 76.0 | A (0.005);  B (0.000) | 31.6 ±38.4 | |  |
| T cells (CD3+CD19-) % | 69.5 ± 11.4 | C (0.001) | 69.0 ± 10.2 | C (0.000) | 56.2 ±12.9 |  | 69.5 ± 6.4 | | C(0.001) |
| T cells (CD3+CD19-) /ul | 867 ± 472 | C (0.008) | 955 ± 453 | C (0.000) | 444 ± 321 |  | 742 ± 477 | |  |
| Ts cells (CD3+CD8+) % | 24.7 ± 5.9 |  | 27.2 ± 28.2 |  | 19.5 ± 6.7 |  | 24.8 ± 9.5 | |  |
| Ts cells (CD3+CD8+) /ul | 317 ± 145 | C (0.002) | 331 ± 159 | C (0.000) | 148 ± 97 |  | 333 ± 212 | | C(0.001) |
| Th cells (CD3+CD4+) % | 38.5 ± 7.6 |  | 37.9 ± 9.3 | C (0.007) | 31.8 ± 11.3 |  | 35.2 ± 8.5 | |  |
| Th cells (CD3+CD4+) /ul | 508 ± 272 | C (0.003) | 490 ± 283 | C (0.000) | 232 ± 158 |  | 378 ± 252 | |  |
| NK cells (CD3-/CD16+CD56+) % | 13.3 ± 7.8 |  | 15.0 ± 9.6 |  | 22.3 ± 13.6 | A (0.032);  B (0.002) | 14.8 ± 7.3 | |  |
| NK cells (CD3-/CD16+CD56+) /ul | 142 ± 87.8 |  | 20 ± 165 |  | 163 ±114 |  | 172 ± 112 | |  |
| B cells (CD3-CD19+) % | 14.0 ± 5.8 |  | 13.0 ± 4.9 |  | 17.7 ± 9.4 | B(0.001);  D(0.023) | 11.7 ± 4.5 | |  |
| B cells (CD3-CD19+) /ul | 178 ± 132 |  | 183 ± 113 | C (0.013) | 121 ± 79.7 |  | 130 ± 82.3 | |  |
| Th/Ts | 1.63 ± 0.43 |  | 1.78 ± 0.85 |  | 1.85 ± 1.01 |  | 1.48 ± 0.82 | |  |
| Lymphocyte count (× 10^9^/L) | 1.27 ± 0.50 | C (0.002) | 1.35 ± 0.56 | C (0.000);  D(0.012) | 0.78 ± 0.41 |  | 0.99 ± 0.44 | |  |

Note: S and G stand for chest CT performance of the patients. S: multiple small spot shadow; G: multiple ground glass shadow or infiltrative shadow.

**Table S4 Gains for the terminal nodes in the model (decision tree model with chest CT）**

| Node | Node | | Gain |  | Response | Index |
| --- | --- | --- | --- | --- | --- | --- |
|  | N | Percent | N | Percent |  |  |
| 6 | 24 | 10.8% | 17 | 53.1% | 70.8% | 491.4% |
| 8 | 55 | 24.8% | 14 | 43.8% | 25.5% | 176.6% |
| 4 | 7 | 3.2% | 1 | 3.1% | 14.3% | 99.1% |
| 3 | 131 | 59.0% | 0 | 0.0% | 0.0% | 0.0% |
| 7 | 5 | 2.3% | 0 | 0.0% | 0.0% | 0.0% |

**Table S5 Confusion matrix analysis for decision tree model with chest CT**

| Observed | Predicted | |
| --- | --- | --- |
|  | Mild or common cases at admission | Severe or critical cases at admission |
| Mild or common cases at admission | 183 | 7 |
| Severe or critical cases at admission | 15 | 17 |

**Table S6 Gains for the terminal nodes in the model (decision tree model without chest CT）**

| Node | Node | | | Gain | |  | | Response | | Index |
| --- | --- | --- | --- | --- | --- | --- | --- | --- | --- | --- |
|  | N | Percent | N | | Percent | |  | |  | |
| 5 | 21 | 9.5% | 11 | | 34.4% | | 52.4% | | 363.4% | |
| 8 | 16 | 7.2% | 7 | | 21.9% | | 43.8% | | 303.5% | |
| 6 | 26 | 11.7% | 6 | | 18.8% | | 23.1% | | 160.1% | |
| 7 | 135 | 60.8% | 8 | | 25.0% | | 5.9% | | 41.1% | |
| 3 | 24 | 10.8% | 0 | | 0.0% | | 0.0% | | 0.0% | |

**Table S7 Confusion matrix analysis for decision tree model without chest CT**

| Confusion matrix (for decision tree model without chest CT） | | |
| --- | --- | --- |
| Observed | Predicted | |
|  | Mild or common cases at admission | Severe or critical cases at admission |
| Mild or common cases at admission | 180 | 10 |
| Severe or critical cases at admission | 21 | 11 |

**
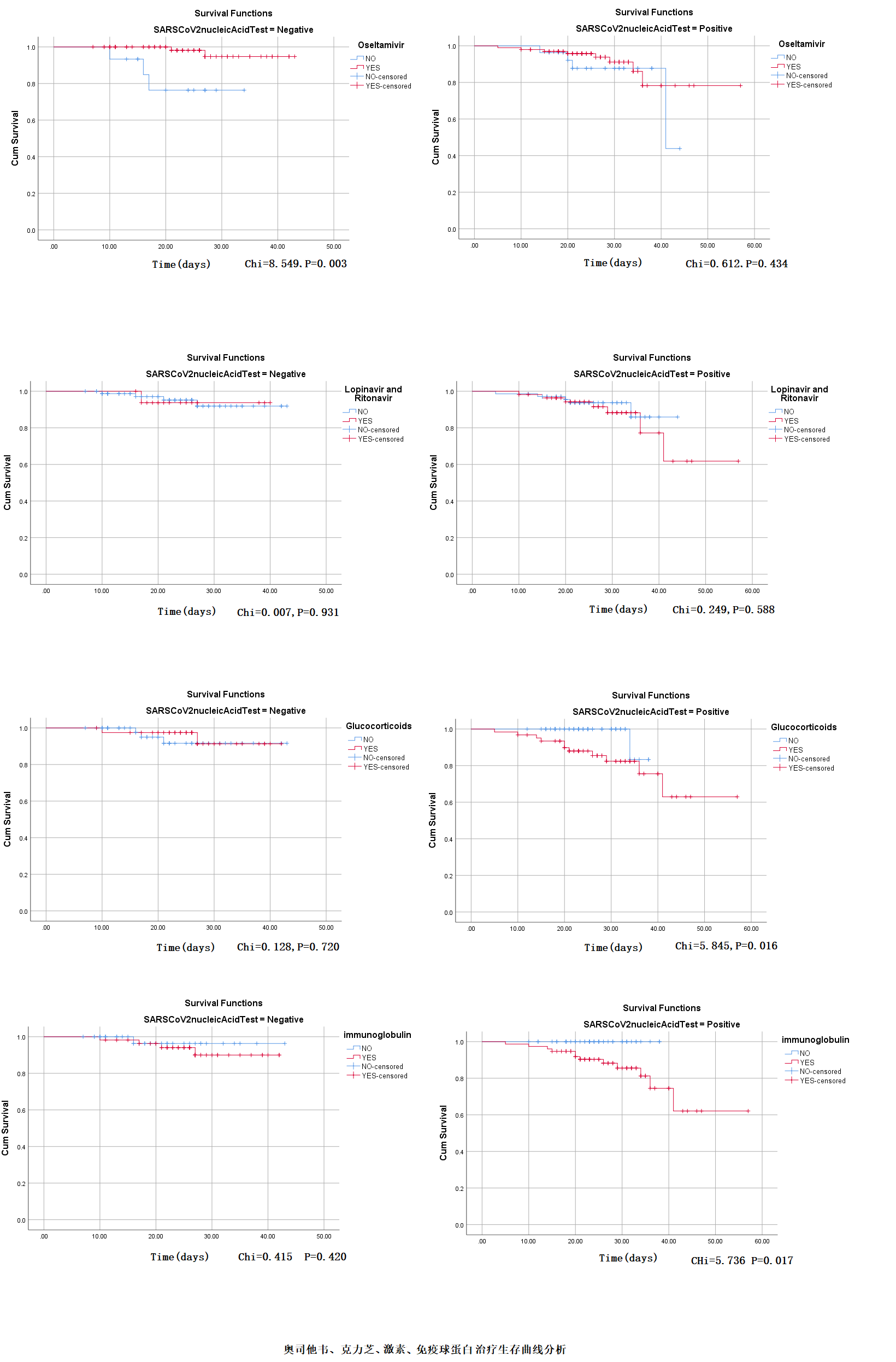
**

**Figure S1 Survival curve analysis of treatments with oseltamivir, lopinavir and ritonavir, and glucocorticoids**


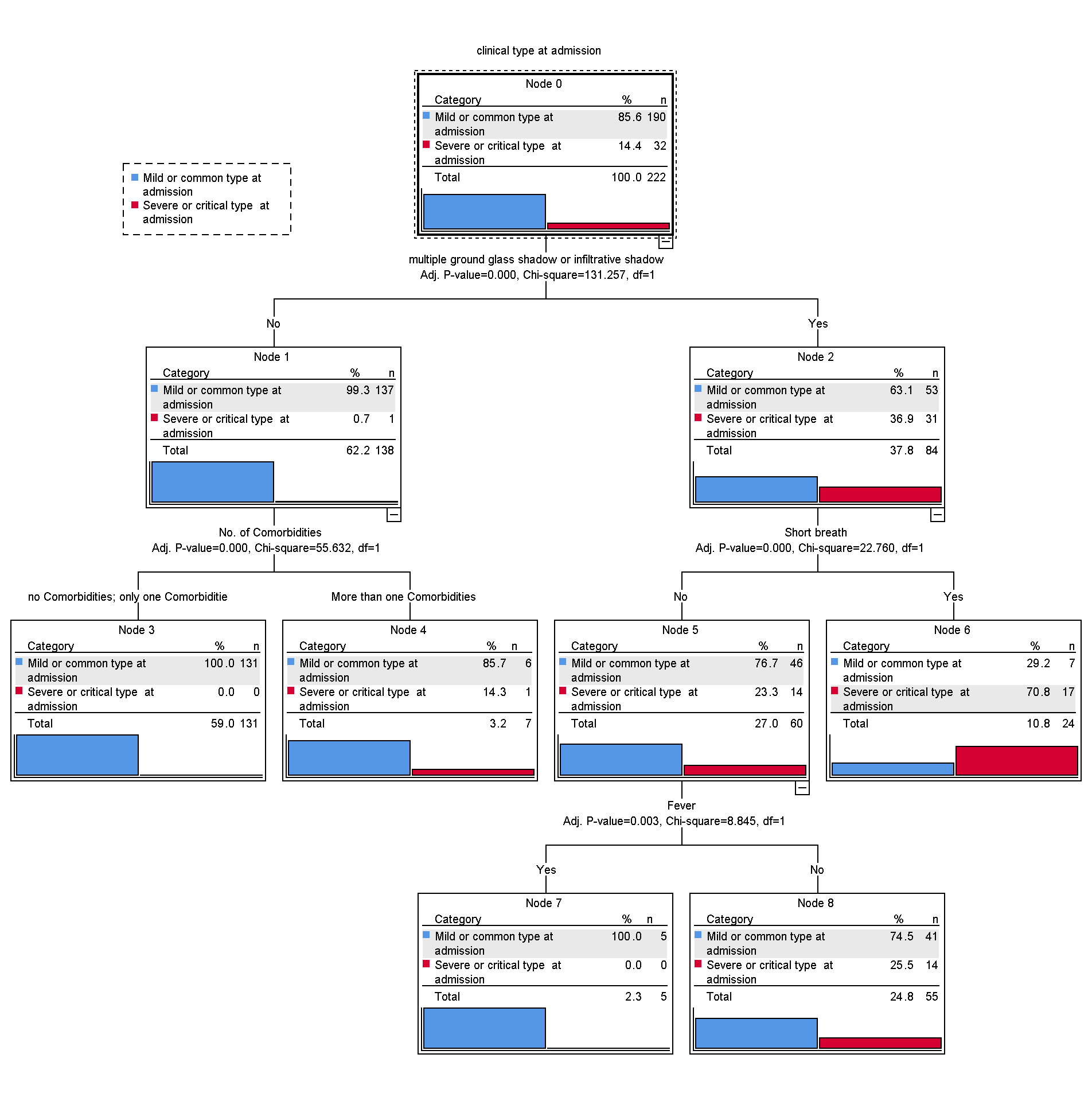


**Figure S2 Clinical decision tree to determine a severe or critical case with chest CT scan**


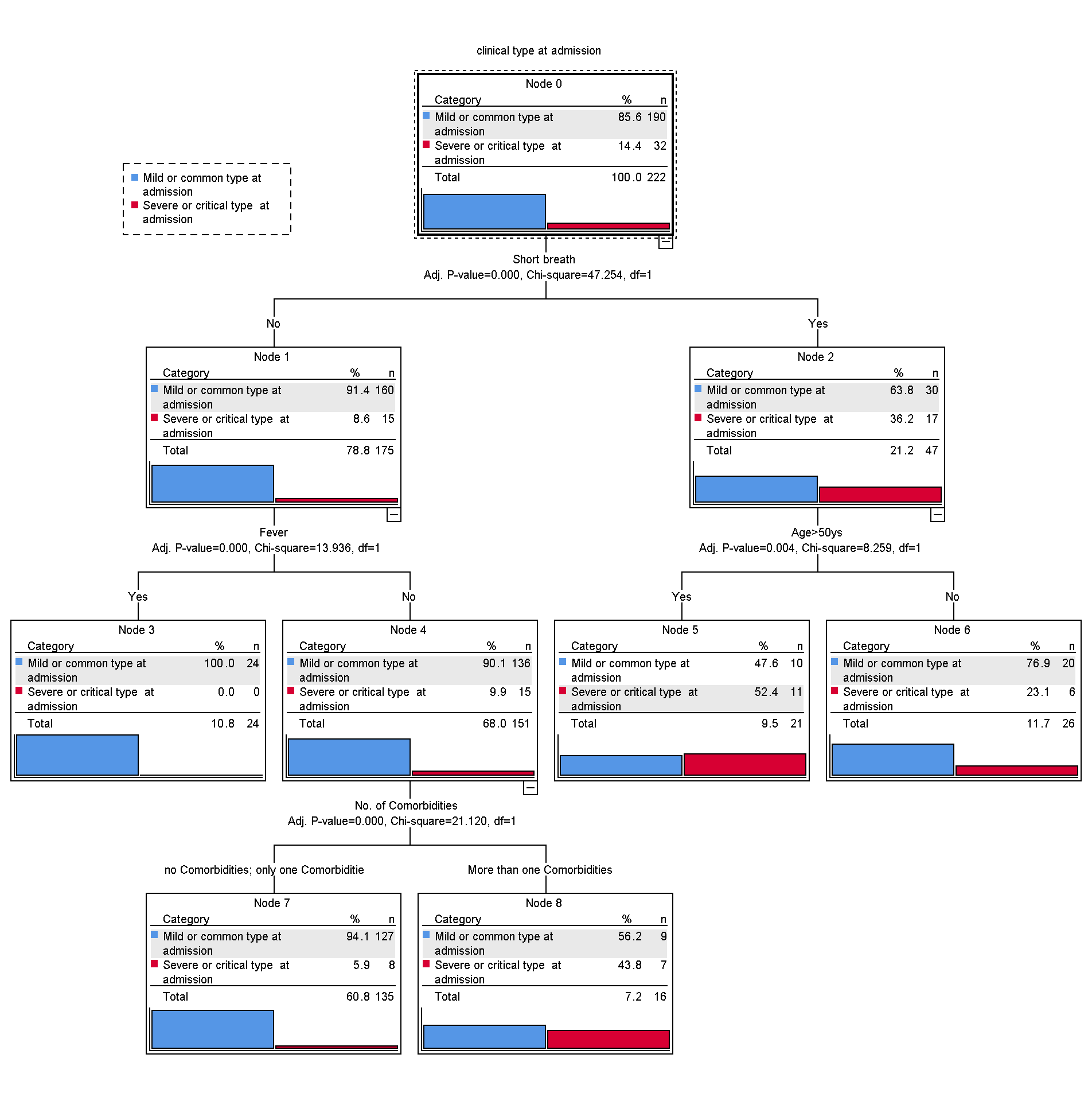


**Figure S3 Clinical decision tree to determine a severe or critical case without chest CT scan**
